# Autogenic spinal excitatory circuit ensures skilled hand movements in primates

**DOI:** 10.1073/pnas.2525051123

**Published:** 2026-03-19

**Authors:** GeeHee Kim, Saeka Tomatsu, Tatsuya Umeda, Tomohiko Takei, Tetsuro Funato, Kazuhiko Seki

**Affiliations:** ^a^Department of Neurophysiology, National Institute of Neuroscience, National Center of Neurology and Psychiatry, Tokyo 187-8502, Japan; ^b^Department of Developmental Physiology, National Institute for Physiological Sciences, Aichi 444-8585, Japan; ^c^Department of Mechanical Engineering and Intelligent Systems, The University of Electro-communications, Tokyo 182-8585, Japan

**Keywords:** nonhuman primates, spinal interneurons, proprioceptive feedback, voluntary hand control, computational modeling

## Abstract

Skillful hand movements are a hallmark of primates, including humans, requiring sophisticated motor planning and execution. Building on the well-established cortical basis of dexterous control, our findings show that spinal excitatory reflex circuits form a critical complementary pathway that contributes substantially to the planning and execution of skillful hand movements. Using a combination of experimental approaches with behaving nonhuman primates and predictive simulation, we identified a group of excitatory spinal interneurons that orchestrate a closed-loop, positive feedback mechanism during voluntary wrist movements. This mechanism is characterized by a bidirectional interaction between interneuronal spiking and muscle activity, mediated by motoneuronal efferent signals and proprioceptive afferent signals from the same agonistic muscles. Furthermore, we demonstrate that the temporal profile of muscle activity during movement execution, including amplitude and duration, is predetermined during motor planning at the spinal interneurons, functioning as a force-feedback gain within the excitatory circuit. These findings suggest that autogenic, Ib spinal excitatory circuits play a predominant role in shaping overall muscle activation during motor execution, provided the proper reflex gain is preset by higher neural systems during motor planning. Together, our findings provide cellular-level evidence that spinal reflex loops operate in parallel with cortical mechanisms to support skilled voluntary movements in primates.

## Significance

Skilled hand movements are a hallmark of primate behavior and are usually attributed to cortical mechanisms. Yet the degree to which spinal circuits contribute directly to voluntary dexterity has remained unclear. By combining in vivo recordings, peripheral stimulation, and computational modeling, we identify an excitatory spinal circuit in macaques that forms an autogenic positive feedback loop: spinal interneurons receive proprioceptive input from hand muscles and in turn provide excitatory drive back to those muscles. This loop generates task-related activity tightly coupled to muscle output, contributing to cortical control of skilled hand movements via spinal reflex pathways. These findings preclude any view of the spinal cord as a passive relay and highlight its role in volitional motor control and rehabilitation strategies.

The spinal cord, which is intricately connected to the brain, plays a crucial role in coordinating various body movements, and is especially significant in controlling stereotyped actions, such as locomotion and postural control. Within the spinal cord, segmental reflex circuits constitute the core modules for sensorimotor transformations during movement. These reflex circuits, present in diverse species, from invertebrates to higher mammals, including humans, directly, efficiently, and quickly integrate somatosensory signals into ongoing motor actions. Physiological studies (1, 2) and recent advancements in genetic tools (3, 4) have led to the characterization of several reflex circuits and their key interneurons, delineating their distinct input–output properties. Despite extensive evaluation in relation to stereotyped movements (5), the involvement of these circuits in the execution of nonstereotypical movements (6), such as skilled voluntary movements, remains largely unexplored.

Conceptually, muscle activation during skilled voluntary movement has been discussed in terms of how cortical commands are routed through spinal motor circuits. Historically, dominant frameworks emphasized relatively direct corticospinal pathways, in which motor cortical activity can influence motoneurons and muscles with minimal spinal processing (7–11). However, our recent studies demonstrated that pathways acting predominantly through spinal interneuronal networks play an important role in shaping muscle activation (12–15). This involvement of spinal interneurons in the control of volitional movement is further supported by the work in nonprimate species—including cats (16), rats (17–19), and mice (20)—demonstrating that many learned and skilled motor behaviors can persist after motor cortex lesions. These results imply that cortical contributions may be complemented by subcortical and spinal mechanisms.

Here, we hypothesized that spinal reflex circuits represent an essential, jointly operating mechanism that works together with cortical pathways to support muscle activation during voluntary movement in nonhuman primates. This hypothesis was investigated based on the spinal excitatory interneuronal activity that mediates voluntary muscle activation in behaving monkeys, and we modeled their implementation in the neural system that controls voluntary motor action.

## Results

**Fig. 1.**
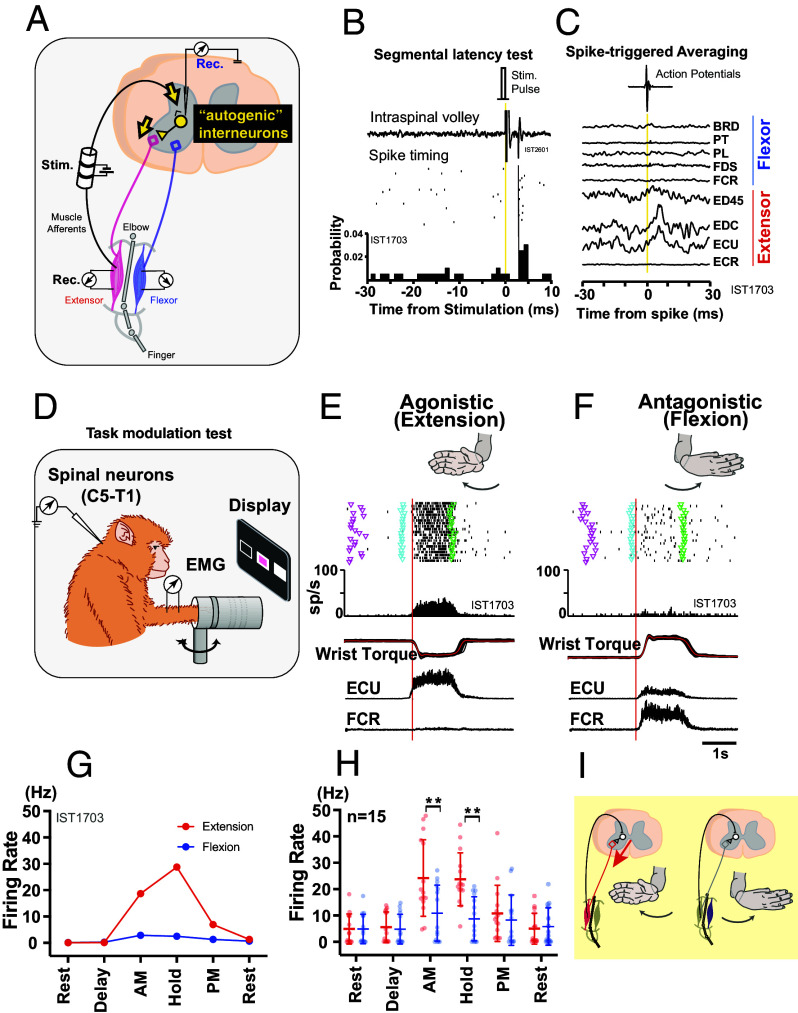
Excitatory interneurons mediating autogenic facilitation of wrist-extensor muscles. (*A*) A method to identify the interneurons mediating autogenic excitatory segmental spinal reflexes (“autogenic” interneurons). The afferent nerve innervating the wrist extensor muscles (deep radial nerve, DR nerve) was stimulated using an implanted nerve cuff electrode (Stim), and neurons showing a response with a monosynaptic latency were identified (see *B* for details). The output connections of the same neurons to motoneurons of the wrist flexor and extensor muscles were then determined by averaging electromyographic (EMG) signals recorded from the wrist flexor and extensor muscles (Rec) (see *C* for details), and among these, neurons showing a significant postspike facilitation in the wrist extensor muscles were defined as “autogenic” interneurons. Arrows indicate the input to and output from the interneurons. (*B*) Examination of afferent input to the interneurons. A representative neuron showing a monosynaptic response to stimulation. Neurons showing responses to the electrical stimulation (“stim. pulse”, yellow line) with a central latency of less than 1.5 ms relative to the volley (black line), as evaluated by the peristimulus time histogram (PSTH, *Bottom*), were deemed first-order interneurons. (*C*) Examination of interneuron output connections to motoneurons by spike-triggered averaging. EMG signals recorded from forearm muscles were averaged after being triggered by the timing of each action potential of the interneuron (*Top*, yellow line). Shown is the same interneuron as in *B*. Neurons showing significant postspike facilitation in wrist extensor muscles (EDC and ECU) were deemed premotor interneurons. Based on the input (*B*) and output (*C*) profiles, this example neuron was identified as an excitatory interneuron mediating afferent input from wrist extensor muscles and projecting to motoneurons innervating wrist extensor muscles (EDC and ECU) (“autogenic” interneurons). BRD, brachioradialis; PT, pronator teres; FDS, flexor digitorum superficialis; FCR, flexor carpi radialis; ED45, extensor digitorum-4,5; EDC, extensor digitorum communis; ECU, extensor carpi ulnaris; ECR, extensor carpi radialis. (*D***–***F*): Evaluation of the firing patterns of the identified interneurons in monkeys performing voluntary movements. While the monkeys performed wrist flexion and extension tasks with an instructed delay period (*SI Appendix*, Fig. S1), the interneuron activity was recorded (*D*). (*E* and *F*) Example activity of an autogenic interneuron (the same neuron as in *B* and *C*) during wrist extension (agonistic to the DR nerve, *E*) and flexion (antagonistic to the DR nerve, *F*) trials. From *Top* to *Bottom*: dot raster plot, perievent time histogram, wrist torque (+, flexion; −, extension; the red line indicates the averaged torque trace), and averaged EMG traces of ECU and FCR. Each sweep was aligned to the onset of wrist torque (red line). Data from 21 flexion and extension trials are shown. Triangles indicate the timing of task start (magenta), first “go” cue (blue), and second “go” cue (green) (see also *SI Appendix*, Fig. S1). (*G* and *H*) Modulation of the firing rates of autogenic INs during the task. (*G*) Mean firing rate in each task epoch of the same interneurons *B*, *C*, *E*, and *F*. Delay: Instructed delay period, AM: active movement. Hold: Active hold period; PM: passive movement period. (*H*) Mean ± SD of the firing rate of all agINs (n = 15; *SI Appendix*, Fig. S2). **P < 0.01. (*I*) Schematic description of the input–output relationship of agINs and corresponding firing pattern during wrist extension (*Left*) and flexion (*Right*) tasks.

We trained three monkeys to perform wrist flexion-extension tasks (21) (*SI Appendix*, Fig. S1), which constitute nonstereotyped sensorimotor behavioral tasks because the monkeys need to adjust the kinematics and dynamics of their wrist movements according to varying visual instructions. During the performance of the trained task, we recorded the activity of interneurons (INs) which mediate the segmental spinal reflexes (Reflex INs) in the cervical spinal cord. The reflex INs were identified using both input from and output to the periphery (Fig. 1 *A*-*C*). Based on the response to the electrical stimulation of the primary sensory (Group I) afferents of the wrist extensor muscles (deep radial nerve (DR), Fig. 1*A*), input was examined (Fig. 1*B*) (21, 22) whereas the output was determined by spike-triggered averaging (STA) of reflex INs’ effect on the motoneuron pool of the wrist and finger muscles (Fig. 1*C*) (12, 23). Of the 292 neurons recorded in the monkeys, 35 reflex INs were identified (*SI Appendix*, Fig. S2*A*) and were categorized into six possible input–output patterns based on their target muscle (*SI Appendix*, Fig. S2*B*). These reflex INs predominantly evinced an “autogenic” pattern (i.e., INs receiving Group I afferents from wrist-extensor muscles and also projecting to the wrist-extensor motoneurons, n = 20/35), and a majority (75%) were excitatory IN (*SI Appendix*, Fig. S2*B*). Excitatory INs that mediated autogenic reflexes (n = 15) were named “agINs” (Fig. 1*A*). The representative activity of agINs during voluntary wrist movements (Fig. 1*D* and *SI Appendix*, Fig. S1) is shown in Fig. 1*E* (wrist extension) and Fig. 1*F* (flexion; the same neurons shown in Fig. 1 *B* and 1 *C*). The agIN firing pattern was highly biased toward wrist extension (Fig. 1*G*). Among the 15 agINs, 13 showed extension-biased activity, one showed flexion-biased activity, and one showed an unbiased activity pattern, indicating a highly significant bias toward extension (*P* < 0.001, binomial test); the grand averages of these 15 agINs also exhibited extension-biased activity (*P* = 0.002, two-way ANOVA, Fig. 1*H*).

**Fig. 2.**
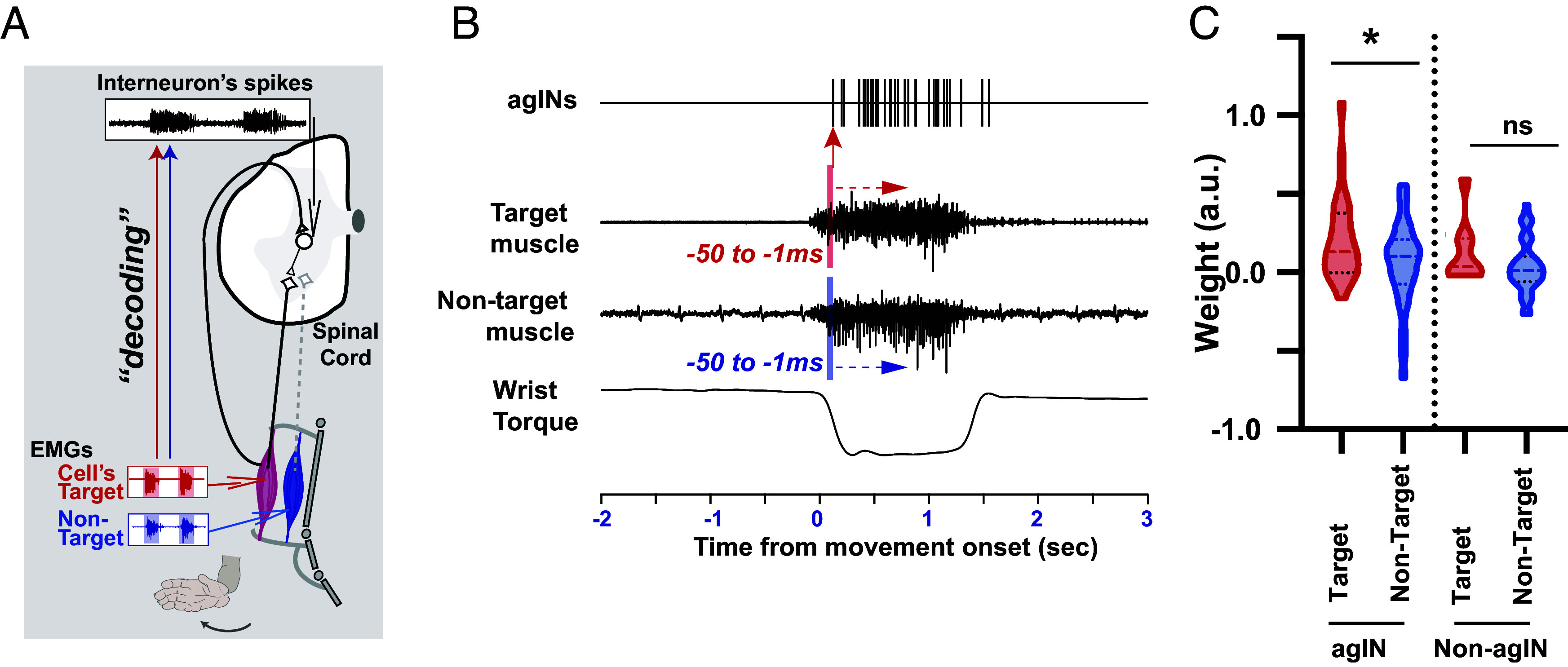
Decoding agIN and non-agIN firing patterns from muscle activity (*A*) The supposed closed loop consists of agINs, their target extensor motoneurons (black diamonds) and muscles (red), and sensory afferent fibers directly projecting to the same agINs. Nontarget agonistic motoneurons (gray diamonds) and muscles (blue) are also illustrated for comparison. The capability of the activity of each agIN’s target muscle to affect the spiking activity of agINs during agonistic movement (*Bottom*, wrist extension) was examined by comparing the decoding performance of agIN activity using the EMG of the target muscle (red) and nontarget muscle (blue). (*B*) Details of the decoding analysis. For each agIN, the influence of the extensor muscle activity on its future spiking activities (≤50 ms) was examined by comparing the contribution of the EMG of the target muscle and that of the nontarget muscles to the decoding of agIN activities using the EMG of the target muscle (red arrow) and that of the nontarget muscle (blue arrow). See *Materials and Methods* for details. (*C*) *Left*, weight values of the EMG of the target (red) and nontarget (blue) extensor muscles in models that predicted agIN activity. *P < 0.05 (Unpaired *t* test). *Right*: same comparison in non-agINs.

**Fig. 3.**
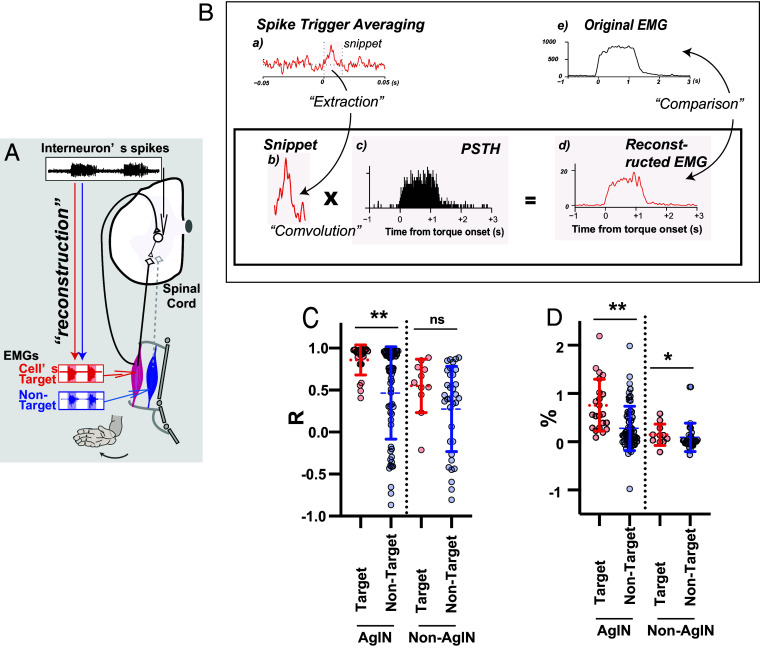
Reconstruction of muscle activity from agIN and non-agIN output profiles (*A*) The contribution of agINs to generating EMG signals in their target muscle was examined using a “reconstruction” analysis. See the legend for Fig. 2*A* for details. (*B*) Procedure for the reconstruction analysis used to evaluate the contribution of single agINs to the generation of EMG signals in target muscles. (*a* and *b*) Waveform of postspike facilitation of the target muscle (ECU) for a single agIN. Shading indicates the snippet extracted from the waveform and used for the analysis (15 ms from the trigger). (*c*) Firing pattern (PETH) of the same agIN as *a* and *b*, aligned to movement onset. Bin width = 5 ms. Data were compiled from 51 extensor trials. The snippet (*b*) extracted from the waveform obtained by the spike-triggered average (*a*) was convolved with the PETH to obtain the reconstructed EMG (*d*), which was then compared with the original EMG (*e*) of the target muscle (same muscle as *a*), smoothed and averaged across 51 extensor trials. Note the difference in the vertical scale between *d* and *e* (see main text). (*C*) *Left*, correlation coefficients between the original EMG (as in *B, e*) and reconstructed EMG (as in *B, d*) of the target (red) and nontarget (blue) muscles of the agINs. Dotted lines indicate means and continuous lines indicate ±SD, *P* < 0.01. *Right*, same comparison for non-agINs. (*D*) Comparison of the amplitude of the reconstructed EMG relative to the original EMG, shown in the same format as in *C*.

Based on the characteristic input–output pattern of agINs, they could be identified as the “Ib INs” (2, 24), which mediate the Group I oligosynaptic- (25) or disynaptic (26, 27) input to the extensor motoneuron from both Ia and Ib afferents (2, 28, 29). In “reflex reversal,” the Ib INs have been found to become excitatory during locomotion (26, 30), while otherwise suppressing synergistic motoneurons (31). We hypothesized that the agIN’s predominant activity during extension reflects their unique role in controlling voluntary movements by generating and modulating agonistic muscle activity (Fig. 1*I*) through Group Ib afferents (32), which are activated predominantly via a recurrent positive-feedback reflex loop involving the same agonistic muscles. However, while Fig. 1 establishes the characteristic synaptic input–output profile of agINs and their extension-biased recruitment during the task, it does not by itself determine whether the proposed loop is functionally effective—i.e., whether ongoing extensor muscle activity can robustly drive agIN firing, and whether agIN activity, in turn, can account for the task-related pattern of extensor EMG. Therefore, to evaluate the functional coupling implied by this circuitry, we tested this hypothesis by examining whether 1) the activity of the extensor muscle during wrist extension effectively influences the activity of agINs (Fig. 2*A*, *decoding*), and 2) the activity of agINs can reproduce the task-related extensor EMG pattern (Fig. 3*A*, *reconstruction*).

First, using electromyographic (EMG) signals of the wrist muscles, we decoded the agIN firing activity during wrist extension. This analysis focused on the 50- to 1-ms duration preceding the occurrence of each agIN action potential (Fig. 2*B*) with the rationale that, if agIN firing is partly driven by sensory afferent signals from their target muscles, a higher decoding weight could be expected using EMGs of their target muscles (red arrow in Fig. 2 *A*, *Left*), rather than of the synergistic, nontarget muscle (blue arrow). As each agIN receives a direct projection from the extensor afferent (Fig. 1*B*), this analysis could determine whether the extensor muscle’s activity drives agINs through this projection. The agIN firing profile was successfully predicted by the EMG of the target and nontarget muscles, both at the single-test level (*SI Appendix*, Fig. S3*A*) and population level (*SI Appendix*, Fig. S3*B*). Furthermore, we found a higher decoding weight in the agIN’s target muscle (n = 22) than that of their nontarget synergistic muscles (Fig. 2 *C*, *Left*, n = 29, *P* = 0.045, unpaired *t* test). A comparable result in the non-agINs (i.e., reflex INs with positive postspike effect on the flexor muscles, “heterogenetic” and “both” neurons in *SI Appendix*, Fig. S2) between the target and nontarget muscles (Fig. 2 *C*, *Right*) was not identified, which suggests that the observation is agIN specific.

These decoding results support the conclusion that the afferent input pathway suggested by Fig. 1 is functionally effective during voluntary wrist extension: extensor muscle activity—particularly from each agIN’s target muscle—provides the dominant drive for agIN spiking, consistent with the recruitment of group I afferent input to agINs. Importantly, we do not claim that sensory afferent input is the sole source of drive for agIN spiking during voluntary wrist extension. A substantial fraction of their activity could also reflect descending cortical commands transmitted through corticospinal pathways (33). In line with this, agIN spiking could be predicted from EMG with relatively high, but incomplete, accuracy, consistent with a mixture of sensory and descending influences. Critically, however, the decoding weights were systematically higher for EMG from each agIN’s target extensor muscle than for EMG from nontarget synergists, indicating a preferential functional coupling to the target muscle rather than a nonspecific relationship to overall wrist muscle activation. Moreover, because reafferent signals routed through the transcortical sensorimotor loop would be too delayed to influence agIN firing within the short time window relevant for our decoding (∼50 ms preceding the spike) (34), this target-selective coupling is more consistent with fast peripheral pathways, supporting effective recruitment of the direct group I afferent input to agINs during wrist extension.

Next, to assess the output aspect of this functional coupling, we asked whether agIN activity can drive the task-related extensor EMG of their target muscles during wrist extension. Specifically, we quantified the extent to which each agIN’s spiking—together with its measured postspike facilitation, estimated by the STA—can reconstruct the EMG time course of the target muscle (Fig. 3*A*, “reconstruction”) (15).

First, we compiled a snippet from the STA of both target and nontarget muscles (Fig. 3 *B*, *a* and *b*) that reflects the contribution of a single spike of agINs to generating muscle activity (7, 9). In parallel, we calculated the perievent time histogram (PETH) of spikes for each agIN around the movement onset (Fig. 3 *B*, *c*). By calculating the convolutions of each PETH and snippet (Fig. 3 *B*, *b* and *c*), we reconstructed the EMG signals of each muscle (Fig. 3 *B*, *d*), and compared them with the original EMG signal of the same muscle (Fig. 3 *B*, *e*). In this example (See *SI Appendix*, Fig. S4 for another example), the reconstructed EMG exhibits a temporal profile that correlates with the original EMG (from onset to offset; n = 10,383 points, R = 0.966). We further compared the magnitudes (total area from onset to offset of the original EMG) of the reconstructed and original EMG signals by calculating their ratios. The ratio in the example (1.55% of the total area of the original EMG) was greater than those of three nontarget extensor muscles of this agIN [0.39 ± 0.28% (SE; n = 3; individual values: 0.10, −0.005, 1.09)].

We repeated the same analysis for all agINs and excitatory non-agINs. The similarity with the original EMG profile was higher when the reconstructed EMG was created from the snippet of the target muscle (R = 0.86 ± 0.03, n = 22) than of nontarget muscles in agINs (R = 0.46 ± 0.07, n = 61, *P* < 0.01, unpaired *t* test, Fig. 3 *C*, *Left*). We also found that the excitatory non-agINs showed much lower similarity of the reconstructed EMG with their target muscles [Fig. 3 *C*, red on the *Right* and *Left*, R = 0.86 ± 0.03 (agINs) vs. 0.55 ± 0.09 (excitatory non-agINs), n = 22 vs. n = 11, *P* < 0.01, unpaired *t* test]; furthermore, the excitatory non-agINs showed indifferent similarity in the EMG reconstructed either by their target or nontarget muscles [Fig. 3 *C*, *Right*, R = 0.55 ± 0.31 (target muscles) vs. 0.27 ± 0.50 (nontarget muscles), n = 11 vs. n = 34, *P* = 0.10, unpaired *t* test]. We also found that the single agIN could generate 0.75% (±0.12%) of the original EMG, which is larger than the reconstructed EMG of nontarget muscles (Fig. 3 *D*, *Left*, 0.27 ± 0.05%, n = 22 vs. n = 61, *P* < 0.001, unpaired *t* test). Thus, if we assume that only agINs generated the EMGs, we infer that the original EMGs of the agIN target muscle could be generated by only 114 to 158 (on average, 133) agINs. This number represents the exclusive capability of agINs for motoneuronal activation, because thousands of spinal (35) and nonspinal (36, 37) premotor neurons could mediate EMG generation (15). In contrast, the reconstructed EMGs of the excitatory non-agINs’ target muscles were smaller [size = 0.75 ± 0.11% (agINs) vs. 0.14 ± 0.06%, n = 22 vs. n = 11, *P* < 0.01, unpaired *t* test], and showed little differences between the excitatory non-agINs’ target or nontarget muscles (Fig. 3 *D*, *Right*, 0.14 ± 0.06% vs. 0.09 ± 0.05%, n = 11 vs. n = 34, *P* = 0.58, unpaired *t* test). These results suggest that agINs mediate online muscle-activity augmentation during voluntary movement.

Overall, the combined decoding (Fig. 2) and reconstruction (Fig. 3) results support that the synaptic input–output profile identified in Fig. 1 translates into functionally effective coupling between agINs and their target extensor muscles during wrist extension, consistent with the proposed positive-feedback loop via the Ib-reflex pathway. We found that this loop can account for a substantial portion of the target muscle’s activity, compared with pathways mediated by other reflex INs.

As already mentioned, however, it is important to recognize that the actual agIN activity in vivo is shaped by a mixture of descending (feedforward) drive and sensory feedback, consistent with extensive evidence that Ib interneurons receive substantial descending inputs (1, 2). Consequently, the relative contribution and circuit logic of the Ib reflex pathway to volitional muscle activation cannot be isolated from in vivo recordings alone. To address this limitation, we adopted a deliberately simplified modeling strategy: we reduced the descending component to a minimal, single set-point pulse, and asked how much of the reconstructed EMG profile could be generated by the reflex pathway under this constraint. This simplification allowed us to assess the intrinsic capacity of the agIN-mediated positive feedback loop, separate from the mixed drives present during behavior.

**Fig. 4.**
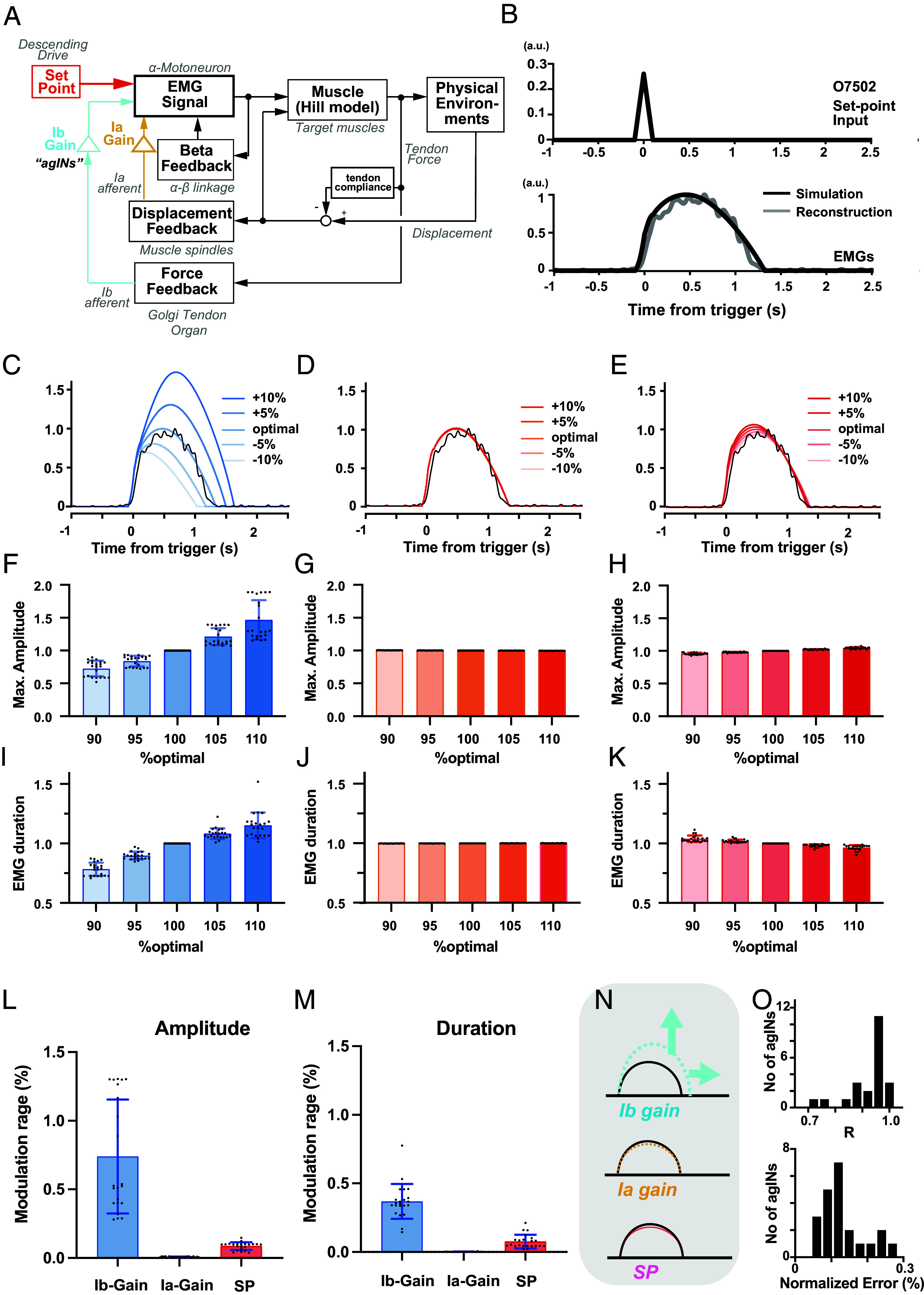
Simulation (*A*) Simplified block diagram of the nonlinear model of the segmental reflex system mediated by the agINs, modified from the existing segmental reflex system model (38). All model parameters, other than the gain parameters, were fixed to the values in the previous report (39–42). See *Materials and Methods* for details. The activity of agINs recruits and augments α-motoneuron activity and generates the EMG in the target muscle. Through the contractile properties and physical environment, the EMG generates autogenic force feedback (via Golgi tendon organs and Ib afferents) and reciprocal displacement feedback (via muscle spindles and Ia afferents) that recurrently modulate the activity of α-motoneurons and target muscles. This model assumes the agINs are driven by the autogenic force feedback (26, 30, 39). (*B*) A reconstructed (gray, n = 89 trials) and simulated (black) EMG of wrist-extensor muscle (ED23) in the extension trial. Simulated EMG was obtained by using the optimal Ib-gain (1.742), Ia-gain (0.871), SP (0.260). R = 0.99. (*C–E*) Example illustrating the effect of different Ib-gain (blue, *C*), Ia-gain (orange, *D*), or SP (red, *E*) on the simulated EMG waveform. (*F–H*) The effects on the maximal amplitude of simulated EMG waveform (n = 21). Note that one neuron-muscle pair has been eliminated from this analysis because of the inability to define the sustaining duration due to the lack of a clear offset in the reconstructed EMGs. (*I–K*) The effects on the duration of the simulated EMG waveform. (*L* and *M*) effects of changing the Ib-gain, Ia-gain, and SP from 90 to 110% of their optimal value on the amplitude (*L*) or the duration (*M*) of reconstructed EMGs. Mean (bar) and SD (line) were also plotted. (*N*) A proposed regulation of EMG burst duration and amplitude by the agINs’ loop. The Ib-gain affects both the amplitude and duration of upcoming EMG burst; neither the Ia-gain nor SP had a significant effect. (*O*) Distribution of the correlation coefficient (*Top*) and normalized difference (*Bottom*) between the simulated and reconstructed EMGs. n = 21.

In an existing model of the segmental reflex system (38), we simulated the EMGs reconstructed from each agIN (Fig. 4*A*). Based on the assumption (Fig. 1) that agINs correspond to “Ib interneurons” (2, 24), which predominantly receives direct input from tendon receptors, we assumed that agINs are solely activated by force-feedback signals from the Golgi tendon organ of the agonist muscle. A remarkable feature of this model is that it can generate time-varying EMG signals that simulate actual reconstructed EMGs with higher accuracy (R = 0.99, Fig. 4*B*) by setting several fixed numerical parameters.

First, we systematically changed the force-feedback gain (Ib-gain, blue in Fig. 4*A*), displacement-feedback gain (Ia-gain, orange in Fig. 4*A*), and the amplitude of the setpoint input (SP; red in Fig. 4*A*), whereas the other model parameters were fixed (*SI Appendix*, Table S1). Representative results are shown in Fig. 4 *C*–*E* (data from the same agINs as in Fig. 4*B* are used). When we increased and decreased the Ib-gain (Fig. 4*C*) around the optimal Ib-gain (providing the best match between the simulated and reconstructed EMG signals), we observed larger and smaller EMG signals (amplitude, n = 5; R = 0.94) with a longer and shorter duration (n = 5; R = 0.91). In contrast, altering the Ia-gain (*D*) or SP (*E*) conferred minimal impact on the simulated EMG signals for amplitude (Ia-gain; n = 5, R = 1.0, SP; n = 5, R = 1.0) or duration (Ia-gain; n = 5, R = 0.87, SP; n = 5, R = 0.96). The same procedure was repeated for 21 agIN–muscle pairs. The results are summarized in *F–K*. When the Ib-gain was increased from 90% to 110% of the optimal value, both the magnitude (Fig. 4*F*) and the duration (Fig. 4*I*) significantly increased (*G*; F = 15.94, *P* = 0.0006; 0.68 ± 0.17 (90% optimal) to 2.22 ± 1.59 (110% optimal), *P* = 0.0015 with Dunnett’s multiple-comparison test, J; F = 10.56, *P* < 0.002; 0.88 ± 0.15 to 1.02 ± 0.07, *P* < 0.011). Increasing either Ia-gain (*G*, *J*) or SP (*H*, *K*) induced subtle or little alteration (Ia-gain, amplitude: F = 16.95, *P* = 0.0005; 1.00 ± 0.00 (90% optimal) to 0.99 ± 0.00 (110% optimal), *P* = 0.0018, duration: F = 1.59, *P* = 0.22; 1.00 ± 0.00 to 1.00 ± 0.00, *P* = 0.53, SP, amplitude: F = 19.06, *P* = 0.003; 0.96 ± 0.04 to 1.04 ± 0.04, *P* = 0.001 with Dunnett’s multiple-comparison test, duration: F = 9.76, *P* = 0.0006; 1.01 ± 0.01 to 0.99 ± 0.01, *P* = 0.0016). The effects of changing the parameter from 90% to 110% of their optimal values were larger in the Ib-gain than in Ia-gain or SP in both the amplitude (Fig. 4*L*, F = 15.03, *P* = 0.0009; *P* = 0.0018 (with Ia-gain) or *P* = 0.0019 (with SP) with Dunnett’s multiple-comparison test) and duration [Fig. 4*M*, F = 12.47, *P* = 0.0019; *P* = 0.0025; with Ia-gain or *P* = 0.0063 (with SP)] of the reconstructed EMGs. This simulation suggested the possibility that the EMG output from the agIN-mediated spinal reflex circuit could be regulated more efficiently by changing the Ib-gain (Fig. 4*N*).

Second, we examined how well the simulation using the optimal Ib-gain predicted the reconstructed EMG signals. As shown in Fig. 4*B*, we observed a high similarity between the actual and simulated EMGs with optimal Ib-gains (Ib-gain =1.867, R = 0.99). We found (Fig. 4*O*) higher correlation coefficients (top, R = 0.91 ± 0.08, mean ± SD) with subtle prediction error (bottom, 0.13 ±0.06% of reconstructed EMG) between the simulated and reconstructed EMG signals from agIN (n = 22). Overall, our model can sufficiently predict the EMG signals of agINs’ target muscles by tuning mostly the single-parameter Ib-gain at the optimal value.

We repeated the same simulation to predict the actual EMG when the agIN activity was recorded (Fig. 1 *G* and *H*), but not the EMG reconstructed from each agIN by changing the Ib-gain, Ia-gain, or SP (*SI Appendix*, Fig. S5). The results were similar to those of the reconstructed EMGs, indicating that the amplitude and duration of the actual EMGs during the task can be sufficiently simulated by changing the Ib-gain (*SI Appendix*, Fig. S5 *A* to *N*) with higher precision (*SI Appendix*, Fig. S5*O*). Taken together, the results of these simulations suggest that the EMG output (amplitude and duration) can be regulated by modulating the Ib-gain of the spinal reflex circuit. In contrast, Ia-gain may be tonically downregulated (43, 44) during wrist extension in this study.

Finally, we confirmed that the input–output gain of agINs (corresponding to the Ib-gain in the simulation) was related to the size and duration of EMG in future actions during voluntary movement. We estimated the trial-by-trial changes in the Ib-gain for each agIN by analyzing the response probability of the agIN to electrical stimuli applied to the muscle afferents of the wrist extensor [Fig. 1*B*, see Confais et al. (22)] before movement initiation (Rest, Cue, and Delay; *SI Appendix*, Fig. S1). We considered the response probability, which could be influenced by both pre- and postsynaptic factors that affect agINs (22), as an indicator of Ib-gain.

**Fig. 5.**
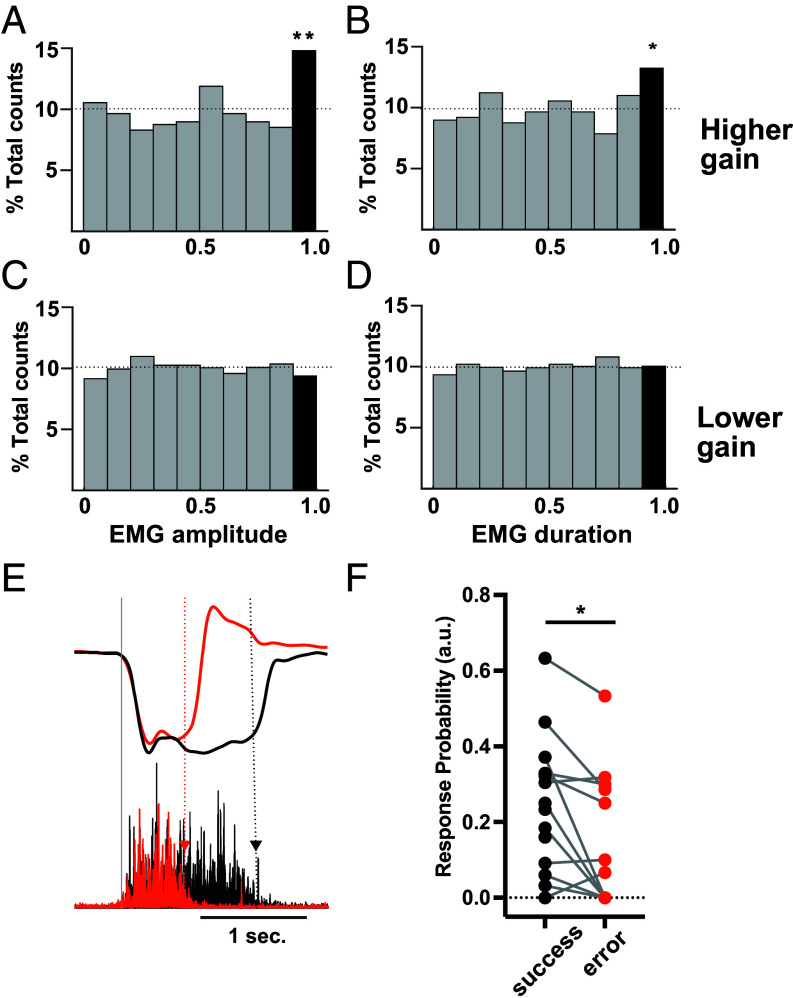
Afferent input gain to agINs and the amplitude and duration of a prospective EMG burst in behaving monkeys (*A* and *B*) Histogram of normalized EMG amplitude (*A*) and duration (*B*) of trials with higher Ib-gain (*P < 0.05, **P < 0.01, binomial test). *C* and *D*: Histogram of normalized EMG amplitude (*C*) and duration (*D*) of trials with a lower Ib-gain. (*E*) Example of a single successful (black) and erroneous (orange) trial. Top: Wrist torque; Bottom: EMG of the agIN’s target muscle (EDC). Gray line: Movement onset. Black and orange dotted lines and triangles indicate EMG offset timing. (*F*) Difference in afferent input gain between successful (black) and erroneous (orange) trials. *P < 0.05.

Our simulation (Fig. 4 *C, F*, and *I* or *N* and *SI Appendix*, Fig. S5 *C*, *F*, and *I* or *N*) indicates the possibility that trials with a high feedback gain to the agIN may induce a large, long-lasting EMG burst. To test this possibility, we compared the normalized amplitude and duration of the EMGs of the target muscles between trials with higher and lower afferent feedback gains. As predicted by the simulation, trials under higher afferent feedback gains exhibited larger amplitudes and longer durations of prospective EMGs (bin > 0.9, binomial test *P* = 0.003 and 0.027, Fig. 5 *A* and *B*) than the baseline probability (dotted line=0.1). In contrast, no statistical bias was observed in trials with lower afferent feedback gains (bin > 0.9, binomial test *P* = 0.119 and 0.768, Fig. 5 *C* and *D*).

Sometimes, monkeys failed the trial when they applied a shorter-duration torque (short-hold, Fig. 5*E*, orange) compared to the successful trial (Fig. 5*E*, black), even though they fully understood the rule of the task. Our simulation predicted that such trial failure might be mediated by a lower Ib-gain. When we specifically compared the response probability of each agIN in the error trials and in the successful trials, we found a smaller Ib-gain before unsuccessful short-hold trials in 75% of agINs (9/12; Fig. 5*F*) and the difference was statistically significant (Fig. 5*F*, 0.25 ± 0.19 vs. 0.15 ± 0.18, *P* = 0.0197, paired *t* test).

Overall, these data analyses of the physiological experiments indicate that regulating the Ib-gain of the segmental reflex pathway plays a functional role in controlling muscle activity and task performance during voluntary wrist movements, which is consistent with the prediction made by the simulation.

## Discussion

**Fig. 6.**
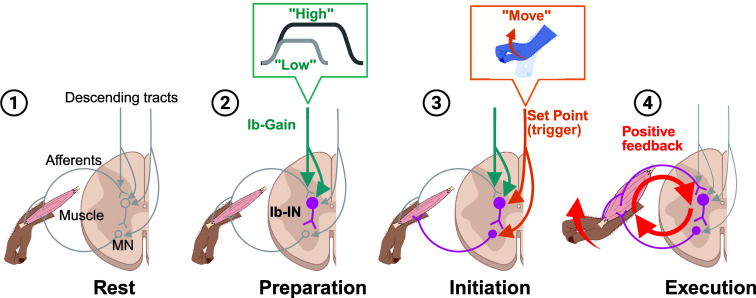
A positive feedback loop mediated by agINs and their mode of action for generating muscle activity during voluntary movement.This figure presents simplified schematics of the positive feedback loop mediated by agINs in different modes of action for generating muscle activity. ➀ *Rest*. No loop activity is observed during this stage. ➁ *Preparation*. The excitability of agINs starts to increase with input from the descending motor command (green arrows), and some agINs may exhibit spiking activity (*SI Appendix*, Fig. S6). However, the excitability of the target motoneurons and muscles remains below the threshold of activation, and thus, no movement is initiated. The excitability of agINs at this stage reflects the duration and magnitude of the prospective EMG of their target muscles encoded by the descending command through increased feedback gain (*Top*, an *Inset*). ➂ *Initiation*. The descending motor command sends a trigger signal to either agINs or their target motor neurons that is strong enough to bring their excitability to a suprathreshold level, and as a consequence, EMG activity is generated. ➃ *Execution*. The EMG activity generated in the initiation state produces a joint torque and force feedback signal (red arrow). Consequently, these feedback signals recurrently activate agINs and their target muscles (red circular arrows) until the duration and magnitude reach the predetermined level during the preparation state. Note that no descending inputs are required during the *execution* state.

The results of the experiments (Figs. 1–3 and 5) and the simulation (Fig. 4) suggest that the positive-feedback reflex system makes a substantial contribution to the generation of muscle activity during voluntary movement. Here, we assessed a simple segmental reflex circuit centered on agINs (Fig. 6, ➀). These interneurons project to motor neurons that activate the muscle whose afferents recurrently excite the same agINs. This circuit also receives descending supraspinal signals.

During the motor preparation period (Fig. 6, ➁), the magnitude and duration of prospective motor action were set within the reflex loop at the agIN level (blue line, descending tract input), which functions as an Ib-gain (blue lines, Fig. 4*A*). It is widely recognized that neurons in the primate primary and premotor cortices exhibit premotor activity, which is correlated with the performance of the upcoming movement (45–47). Given the significant increase of premovement activity observed in some agINs (*SI Appendix*, Fig. S6), these cortical premovement activities, encoding future spinal reflex’s states (33), may be represented in the agINs through corticospinal input (48, 49). This corticospinal neural activity has the capacity to presynaptically and postsynaptically change the Ib-gain to the agINs (2). Moreover, we recently reported that proprioceptive afferent input is facilitated (i.e., Ib-gain increase) before and during agonistic voluntary movement (50) owing to decreased presynaptic inhibition. This premovement activity is unlikely, on its own, to recruit enough motoneurons to initiate joint movement during this motor planning period. First, the overall firing rate during the premovement period in extension trials did not differ from that during the rest period (6.73 ± 6.99 Hz vs. 6.19 ± 6.90 Hz, *P* = 0.45, paired *t* test; Fig. 1*H*). Second, among agINs that showed a significant increase in firing during the instructed delay epoch (5/15 neurons), the firing rate during this period (9.86 ± 7.36 Hz) was slightly higher than their resting rate (6.62 ± 6.43 Hz), but substantially lower than during active movement (36.41 ± 13.40 Hz, *SI Appendix*, Fig. S6*H*). Third, among the agINs that showed increased firing before the EMG onset of their target muscles (6/15 neurons), half exhibited firing onsets within 40 ms of EMG onset, which may contribute to a subthreshold increase in motoneuronal excitability immediately preceding movement initiation (*SI Appendix*, Fig. S6*G*). Rather, these premovement discharges may bias the reflex pathway in a subthreshold manner and are likely accompanied by mechanisms that gate their effective transmission to motoneurons. Such gating would help maintain excitatory–inhibitory balance within spinal circuitry, thereby preventing premature movement onset despite the modest excitation of agINs during movement preparation.

The transient “go” signal from the corticospinal input (13) triggers the looping activity (Fig. 6, ➂, orange lines) by activating either the agINs (2) or the motoneuron innervating the target muscle (9, 51, 52), which serves as the set-point input in the model (Fig. 4*A*). It is important to note that this transient triggering pulse is not intended to represent the actual activity pattern of corticospinal neurons. In physiological conditions, especially during step-and-hold movements such as those studied here, corticospinal neurons typically exhibit phasic or phasic–tonic firing patterns, as widely documented in previous work (e.g., ref. 53) and consistent with our recent recordings in monkeys performing similar tasks (15). In this simulation, however, we deliberately assumed a highly simplified condition in which the corticospinal command serves only as a brief trigger, independent of its usual role in shaping muscle activity. Although such a condition is unlikely to occur in vivo, this simplification allowed us to isolate and examine how the spinal reflex circuit, including the agIN-mediated positive feedback loop, can, by itself, generate muscle activity. Under this simplified condition, the duration and magnitude of the prospective motor action are already embedded within the loop (green lines). Once triggered (Fig. 6, ➃), the looping activity can automatically generate, sustain, and terminate muscle activity without requiring continuous regulation from the corticospinal tract.

These results begin to refine the current understanding of how the sensorimotor cortex and spinal reflex circuits cooperate to control muscle activity during voluntary movement. For decades, cortical mechanisms—particularly those mediated through the corticospinal tract—have been considered central to the generation of volitional motor commands, supported by extensive experimental evidence in monkeys and humans (53–56). Our findings suggest that, under certain conditions, the spinal reflex loop can contribute substantially to the generation of muscle activity, with descending inputs providing essential functions such as setting reflex gain and initiating the looping activity. Rather than acting as an isolated or alternative controller, the spinal and cortical systems likely operate in a coordinated and hierarchical manner to produce skilled motor actions in everyday behavior. Within this framework, we propose that preprogrammed reflex gain enables spinal circuits to generate a fundamental profile of muscle-activity patterns (e.g., gross magnitude and duration), whereas the finer, task-dependent refinements of muscle activity are shaped by the dynamic interaction between cortical and spinal mechanisms (15).

In real-world situations, animals must interact with uncertain environments through intermittent, precise refinements of muscle activity. Such interactions are unlikely to be achieved by the proposed spinal reflex circuit alone, given the preprogrammed magnitude and duration of its prospective muscle outputs. We assume three major roles for descending systems. First, the sensorimotor cortex can function as a predictor (57) of the action of the reflex loop. In this context, its essential role is to set the loop parameter (i.e., Ib-gain) for upcoming movements rather than to generate muscle activity *per se.* Using an updated prediction for the optimal parameter, based on the prediction error from previous experience, the loop parameter is continuously adjusted. Second, and most obviously, the spinal reflex loop needs to be triggered upon movement initiation (corresponding to the “set-point” input in Fig. 4*A*), and the corticospinal projection (52, 54, 58) likely plays the predominant role as this trigger. Third, the cortical mechanism can directly, rather than exclusively through the spinal reflex system, modulate motoneuron firing and muscle activity (34). Such modulation can occur via direct corticomotoneuronal projections or through spinal premotor neurons, including agINs. For example, we recently reported evidence for parallel descending control of finger muscle activity, with more phasic modulation arising from direct corticospinal inputs and more sustained modulation arising from pathways via spinal premotor interneurons (15). These descending pathways provide the most effective means for the online correction of muscle activity in skilled motor output. A critical next step will be to determine how these direct descending influences interact with the reflex-mediated indirect pathway characterized in the present study. Developing models that incorporate both mechanisms will help clarify their respective contributions to flexible motor control.

Based on their gene expression profiles, spinal interneurons have recently been categorized into distinct classes, (59, 60) wherein the subtypes Dl3 and V2a exhibited characteristics similar to those of agINs. Notably, both Dl3 and V2a INs are excitatory and project to the ipsilateral motoneuron pools. The dI3 subtype receives monosynaptic input from proprioceptive group I/II and low-threshold cutaneous afferents, and plays a crucial role in forelimb grasping (61) and hindlimb locomotion (62). Genetic silencing of the dI3 IN output in mice caused loss of grip-strength modulation in response to increasing loads, thus mirroring the role of agINs in maintaining muscle force during hand movements in monkeys (Figs. 4 and 5). In contrast, although direct evidence for Ib-specific afferents remains limited, a subset of Chx10-expressing V2a INs stabilizes and provides ipsilateral hindlimb locomotor activity (63), and their ablation disrupts forelimb-reaching functionality (64). These findings were consistent with the characteristic features of agINs, as illustrated in Fig. 1 and *SI Appendix*, Fig. S2. Though these spinal IN classifications have predominantly focused on rodents and other lower-level animals, bridging the gap between the genetic neuronal characteristics in mice and their roles in regulating voluntary movements in primates has immense potential for advancing our understanding of forelimb motor control.

## Materials and Methods

### Animals.

All experiments were approved by the Institutional Animal Care and Use Committee of the National Institute for Physiological Sciences (NIPS), Okazaki, Japan. We obtained data from three *Macaca fuscata* monkeys [KO and OK (female), and IS (male)]. During the training and recording sessions, each monkey sat upright in a primate chair with its right arm restrained and elbow bent at 90°. The hand of the monkey was held in a cast with the fingers extended and the wrist in the mid-supination/pronation position. The cast holding the monkey’s hand was attached to a servo-motor-driven manipulator that measured the flexion–extension torque about the wrist. The left arm was loosely restrained in a chair (21, 22, 65).

### Behavioral Paradigm.

The monkeys performed a wrist flexion–extension task with an instructed delay period (21, 22, 66, 67) (*SI Appendix*, Fig. S1). The wrist flexion–extension torque applied to the spring-loaded manipulandum controlled the position of the cursor displayed on a computer monitor in front of the monkey. As the monkeys performed the task with their right hand, wrist flexion led to a leftward displacement of the cursor. Trials began with the monkey holding the cursor in a central target window corresponding to zero torque for 0.8 s. Next, the flexion and extension targets (empty rectangles) were shown to the left and right of the central target. One target flashed briefly for 0.2 to 0.5 s (Cue), indicating the correct movement to be performed at the end of the instructed delay period, signaled by the disappearance of the central target (GO). Trials were accepted only if no wrist movements occurred during the delay period (1 s). Following the GO signal, the monkey quickly moved (Active Movement) the cursor to the desired target (<1.5 s, including reaction time) and held the cursor in the target window for 1.5 s (Active Hold). The movements were performed against an elastic load applied using a servomotor (5 N*m). At the end of the Active Hold period, the peripheral target disappeared and the central target reappeared (Release GO). The monkey then relaxed its forearm muscles, allowing the servo spring to passively return the wrist (Passive Movement) to the zero-torque position (Rest). After keeping the cursor within the central target for 0.8 s, the monkey was rewarded with apple sauce (Reward) for successful trials.

### Surgical Procedures.

Following behavioral training, surgeries were performed aseptically after placing the animals under 1.5 to 3.0% sevoflurane anesthesia (2:1 O_2_:N_2_O). Head-stabilization lugs were cemented to the skull with dental acrylic and screw-anchored to the bone. A recording chamber made by stainless-steel or polyetherimide resin (Ultem, Saudi Basic Industries Corporation) was implanted over a hemilaminectomy in the lower cervical vertebrae (C4–C7). Pairs of stainless steel wires (AS631, Cooner Wire) were implanted subcutaneously in 10 to 12 muscles [extensor carpi ulnaris (ECU), extensor carpi radialis (ECR), extensor digitorum communis (EDC), extensor digitorum-2,3 (ED23), extensor digitorum-4,5 (ED45), flexor carpi radialis (FCR), flexor carpi ulnaris (FCU), flexor digitorum superficialis (FDS), palmaris longus (PL), pronator teres (PT), abductor pollicis longus (APL), and brachioradialis (BRD)] that were active in one or both directions (i.e., flexion or extension). Each muscle was identified based on its anatomical location and the characteristic movements elicited by low-intensity intramuscular stimulation. Nerve-cuff electrodes (68) were implanted in the muscle branch (deep radial nerve) and cutaneous branch (superficial radial nerve) of the radial nerve, as well as in the median nerve (22).

### General Recording Procedures.

We penetrated the spinal cord dorsally through an implanted recording chamber with glass-insulated Tungsten or Elgiloy microelectrodes (impedance 0.8 to 1.4 MΩ; Fig. 1*A*) (21). One to three penetrations were performed on each day of recording. When we encountered neurons exhibiting responses to DR stimulation (within the observation window of 10 ms), we measured the segmental latency using the intraspinal volleys as a landmark of (22). Neurons were identified as candidate first-order INs if the response occurred within 5 ms of the stimulus (Fig. 1*B*). We then asked the monkeys to perform wrist flexion–extension tasks and recorded the firing rate modulation as a function of the task epoch for both the flexion and extension trials. For task performance, we examined the output of motoneurons projecting to the wrist flexor and extensor muscles (Fig. 1*C*) by STA (12, 23) (Fig. 1*D*). We identified the recorded spinal neurons as INs by confirming whether they (1) received the putative monosynaptic *input* from DR afferents and (2) sent an *output* to the motoneurons of the wrist and finger muscles by STA. Confirmation of the input–output profile of the recorded neurons was partially performed online but primarily completed postrecording.

### Identification of the Reflex INs.

#### Input to the spinal neurons.

At the beginning of each electrode penetration, intraspinal volleys in the cord surface potentials were monitored (Fig. 1*A*), and the threshold currents that evoked an incoming volley from the DR were measured on most recording days. Subsequently, spinal IN single-unit responses were examined by stimulating the DR with biphasic constant-current pulses (100 µs/phase) at a constant frequency of 1 to 2 Hz. The stimulus current was set at 1 to 1.2 times the threshold. Each action potential was isolated based on its waveform, and peristimulus time histograms (PSTHs) were obtained for each IN. Segmental response latency was calculated from the first peak (69) of the incoming volleys extracted from the cord surface potentials (average of all volleys; Fig. 1 *B*, *Top*) to the onset of the PSTH peak. We adopted a central latency of less than 1.5 ms as the criterion for putative monosynaptic linkage from the DR to neurons identified by the stimulation of each nerve (21, 70, 71).

#### Output from the spinal neurons.

The STA of the rectified EMG was computed to identify the output (postspike effects, PSEs) of spinal INs on the recorded EMGs (12, 14). The STAs were compiled by averaging segments of rectified EMG activity from the 50 ms preceding each trigger to 50 ms after the trigger. Spikes (n > 2,000) were accepted as triggers only if the Rms (RMS) value of the EMG from 30 ms before to 50 ms after the spike was greater than 1.25 times the RMS noise level in the EMG channel. STAs was smoothed using a flat five-point finite impulse-response filter. The baseline trend was subtracted using the incremented-shifted averages method (72), and significant STA effects were identified using multiple-fragment statistical analysis (73) (*P* < 0.00417 for 12 muscles, Bonferroni’s correction). The test window was set to 12 and 3 to 15 ms after the spinal neuron spike. Potential crosstalk between simultaneously recorded EMGs was evaluated by combining a cross-correlation method (7) and a third-order EMG differentiation (74), then STA effects potentially resulting from crosstalk between EMG recordings were eliminated from the present dataset. To distinguish the PSEs from the synchrony effects (75), we measured the onset latency and peak width at half-maximum (PWHM); effects with onset latency >3.5 ms and PWHM <7 ms were identified as PSEs (12, 14). Neurons that showed a large “motor unit” signature in the STA of the nonrectified EMG with only 50 spikes (76) were identified as putative motoneurons and excluded from the dataset.

Neurons confirmed with both monosynaptic input from DR nerve and presumed direct projection to the motoneurons were identified as the INs mediating spinal reflex (Reflex INs in *SI Appendix*, Fig. S2*A*). These neurons were classified according to their output patterns: the target muscles and effect of projection (*SI Appendix*, Fig. S2*B*). When the only target muscles of INs were the wrist extensors, they were classified as autogenic. When those were only wrist flexors, they were classified as heterogenetic. When those were both extensors and flexors, they were classified as both. In contrast, INs with a positive PSE to the target muscles were classified as excitatory, whereas INs with negative PSE were classified as inhibitory.

### Decoding and Reconstruction Analyses.

We examined the functional relationship between agIN activity and muscle activation using EMG-based decoding (Fig. 2 and *SI Appendix*, Fig. S3) and reconstruction (Fig. 3 and *SI Appendix*, Fig. S4) analyses. Preprocessed EMG signals were used to decode agIN firing rates with a sparse Bayesian linear regression framework incorporating short-latency temporal structure. To estimate the contribution of individual premotor neurons to muscle activity, EMG signals were reconstructed by convolving neuronal firing patterns with postspike effect kernels. See *SI Appendix*, *Methods* for detailed descriptions of signal processing, decoding, and reconstruction procedures.

### Positive Feedback Model.

A closed-loop spinal sensorimotor model incorporating force- and displacement-related sensory feedback was used to simulate agIN-generated EMG patterns and to explore how feedback gains shape muscle activation dynamics (Fig. 4 and *SI Appendix*, Fig. S5). See *SI Appendix*, *Methods* for full model description and parameter exploration.

#### Classification and characterization of successful and error trials.

Each trial was considered successful if the monkey completed all the task epochs. Any trial lacking at least one of the task epochs was defined as an erroneous trial. The error trials were classified into three types: 1) *No movement*, trials without any detectable change in torque after the GO signal was delivered. These trials were aborted after the grace period (0.2 to 0.5 s after the GO signal); 2) *Wrong direction*, trials in which movement after the GO signal was opposite to the instructed direction; 3) *Short-hold*, trials in which the monkey moved in the correct direction, but did not hold the instructed wrist torque long enough. The trial was aborted when the torque exceeded or decreased the determined range without returning to the correct range within 0.1 s.

We characterized the EMG signals of the target muscles during successful and erroneous trials by measuring the amplitude and duration of each wrist extension trial. To this end, the EMG of the target muscle was bandpass filtered (50 to 200 Hz) and rectified. The rectified EMG was again filtered using a 5-Hz low-pass filter to emboss the offset of EMG. The onset of EMG was defined as the first time point at which the signal exceeded two SD above the mean value during remaining epoch. The offset of the EMG was defined as the local peak where the value dropped the most sharply at the next local peak. The mean amplitude and duration of the EMG for each trial were measured using the EMG onset and offset time points.

#### Electrophysiological evaluation of afferent feedback gain in each agIN and their relationship with the EMG amplitude and sustained duration of future wrist movement.

We stimulated the DR with biphasic constant-current pulses (100 µs/phase) at a constant frequency of 1 to 2 Hz during the task and measured the evoked response of each agIN during the rest, cue, and delay period in the wrist extension trials (both successful and erroneous trials). For each agIN, the mean evoked response was computed by pooling all the stimulation pulses applied during the task epochs to maximize the signal-to-noise ratio. We then used the onset and offset points of the mean evoked response to compute the peak area evoked in each task period (i.e., using the stimulation pulses applied during a specific period). The peak area represents the firing probability of each agIN for DR stimulation with comparable stimulus intensity. We defined this peak area as the input–output gain of each agIN, as described previously (22).

We performed a trial-based analysis to estimate the trial-by-trial variance of the relationship between the afferent input gain of each agIN and the amplitude and duration of the EMG of the target muscles. For this analysis, we examined whether electrical stimulation elicited a response of agIN activity (spike) within 5 ms of stimuli application during the rest, cue, or delay periods in the wrist extension task (both successful and erroneous trials). Two or three stimuli were typically applied during task epochs. If one of these stimuli elicited the response of agINs, then we defined the trial as the “higher-gain” otherwise as the “lower-gain” trial. Thus, all trials performed during the recording from each agIN, in which at least one stimulus was applied during these task epochs, could fall into two categories.

Next, we measured the maximal amplitude and duration of the EMG of each agIN’s target muscle during higher-gain and lower-gain trials. All EMG signals were bandpass-filtered (50 to 200 Hz), rectified, and low-pass-filtered. The onset of EMG bursts that preceded the movement onset was defined as the starting point, with signals exceeding two SD above the mean EMG amplitude during the rest epoch for at least 50 ms. The offset of EMG bursts that preceded movement offset (defined as the timing of movement onset +1.8 s) was defined as the final point, with signals exceeding one SD above the mean EMG amplitude during the rest epoch for at least 200 ms. The EMG duration was defined as the difference between the timing of EMG onset and offset. The mean EMG amplitude was defined as the mean rectified EMG amplitude between the onset and offset. Subsequently, for each recording day, we sorted the corresponding trials by the mean amplitude (from smaller and larger) or the duration (from shorter and longer) of the EMG bursts and numbered them. For normalization to compile the data on different days, each number was then divided by the number of trials per day. Finally, we compared the distribution of these “relative amplitude” and “relative duration” of the EMG burst of the target muscle between higher-gain and lower-gain trials by making histograms (bin size = 0.1).

#### Onset latency of agIN firing relative to EMG onset.

To quantify the onset latency of agIN activity relative to muscle activation, the following procedure was applied. First, the onset of EMG activity of the extensor carpi ulnaris (ECU) muscle was identified for each successful trial. EMG signals were band-pass filtered (50 to 200 Hz) and rectified, and EMG onset was defined as the first time point exceeding two SD above the mean baseline level. Second, spike trains recorded from each agIN were aligned to the detected ECU onset. Third, spike trains were converted into peristimulus time histograms (PSTHs) with 10-ms time bins, and the firing-onset latency was calculated relative to the ECU onset, which was defined as time zero.

#### Statistical analysis.

All parametric and nonparametric tests used, as well as any multiple-comparison tests and corrections, are indicated in the figure legend or main text, as is the *n*-value for each analysis. All statistical comparisons were two-tailed, when relevant. *P* < 0.05 was considered significant, and * indicates *P* < 0.05 and ** indicates *P* < 0.01; all significant *P*-values are indicated in the figure legends. Statistical analyses were performed using MATLAB or GraphPad Prism version 9.2.0 (GraphPad Software, San Diego, CA).

## Supplementary Material

Appendix 01 (PDF)

## Data Availability

All data have been deposited at GitHub and are publicly available at GitHub: https://doi.org/10.5281/zenodo.18229897 (77). All study data are included in the article and/or *SI Appendix*.
