# Supplementary material for: Autogenic spinal excitatory circuit ensures skilled hand movements in primates
Source: Proc Natl Acad Sci U S A. 2026 Mar 19;123(12):e2525051123. doi: 10.1073/pnas.2525051123 (PMC13012127; doi:10.1073/pnas.2525051123)
Supplement: Supplementary file 1 — Appendix 01 (PDF) [file pnas.2525051123.sapp.pdf]

PNAS

Supporting Information for

Autogenic spinal excitatory circuit ensures skilled hand movements in primates.

GeeHee Kim, Saeka Tomatsu, Tatsuya Umeda, Tomohiko Takei, Tetsuro Funato, Kazuhiko Seki

Kazuhiko Seki, Ph.D.
Email: seki@ncnp.go.jp

This PDF file includes:

Supporting text
Figures S1 to S6
Tables S1
SI References

Supporting Text

Methods.

Decoding agIN activity by EMG signals

First, we preconditioned the EMG and agIN firing rates for the decoding analysis. For the EMG signals, we performed temporal filtering of the EMG signals using a second-order Butterworth high-pass filter (5 Hz). The EMG signals were rectified and computed in 1-ms bins. We calculated the smoothed curves for the signals using a mobile window process with 11 bins. For agINs, we first calculated the instantaneous firing rate by convolving the inversion of the interspike interval with an exponential decay function with a time constant of 50 ms. We computed the firing rate in 1-ms bins, corresponding to the resampling frequency of the EMG.

We then applied a Bayesian Sparse Linear Regression (SLiR) algorithm that introduced sparse conditions for only the channel dimension, and not for the temporal dimension of the model. Using multidimensional linear regression, the instantaneous firing rate of spinal interneurons was modelled as a weighted linear combination of pre-processed EMG signals as follows:

$$y_T(t) = \sum_{k,l} w_{k,l} \times x_{k,T}(t + l\delta) + b \qquad (1)$$

Where $y_T(t)$ is a vector of activity of a spinal interneuron at time index t in a trial T. $x_{k,T}(t+l\delta)$ is an input vector of an EMG signal k at time index t, and time-lag $l\delta$ (δ = 1 ms) in a trial T. $w_{k,l}$ is a weight on an EMG signal k at time-lag $l\delta$, and b is a vector of bias terms to y_T. As we examined how EMG signals influenced the spinal IN activity, time-lag $l\delta$ (Equation 1) was set to negative values. We used EMG signals from −50 ms to time −1 to decode the spinal IN activity at time 0. This shorter lag eliminates the potential involvement of long-loop reflexes (1).

Subsequently, using a training dataset, we built a model to predict the firing rate of spinal INs and tested it using a test dataset. Twenty trials were randomly selected as the training dataset, and one other trial was selected as the test dataset. To assess the model, we calculated the correlation coefficient between the observed and reconstructed spinal IN activities in the test dataset. We performed a 10-fold cross-validation for the analysis of each session and used the average values for the analysis. In the control analyses of model reconstruction, we created surrogate training datasets wherein we randomized the temporal profile of the EMG signals to generate and subsequently test a model.

Evaluation of reconstructed muscle activity from agIN and non-agIN activities

To estimate the contribution of individual premotor neurons to the generation of muscle activity, we reconstructed the EMG signal by convolving neural firing activity (weight) and a post-spike effect waveform (kernel). We used the spike-triggered average (STA) waveform as a kernel.

Specifically, we extracted a time segment of zero to 15ms from the spike onset ("snippet", Fig. 3Bb) from the original time window of the STA (-50ms and + 50ms from the onset) (Fig. 3Ba). In parallel, as a weight of convolution, we calculated the peri-event time histogram (PETH) of spikes for each agIN, from −1000 to 3000 ms from movement onset, with a 5-ms bin. We then calculated the convolution of the PETH and the snippet (Fig. 3B*b,c*) to obtain the reconstructed EMG, which was subsequently filtered with a 10 Hz low-pass (Fig. 3Bd). Raw EMG data were filtered with a 10 –200 Hz band-pass, rectified, aligned by movement onsets for each trial, averaged, and filtered again with a 10 Hz low-pass (Fig. 3B*e*). Finally, we calculated the correlation coefficient (Fig. 3C) between the original EMG (Fig. 3Be) and the reconstructed EMG (Fig. 3Bd). We also calculated the ratio of the area of the reconstructed EMG to that of the original EMG as an index of the relative contribution (% contribution) (Fig. 3D).

Positive feedback model

Diagram

We constructed a modified version of a nonlinear model of the cat hindlimb segmental reflex system (2). In this model (Fig. 4A), the EMG signal at the α-motoneuron is generated by force- and displacement feedback in the closed-loop configurations. An independent drive to the α-motoneuron from outside the closed-loop, emulating the descending motor command ('set-point'), was allocated. To initiate the closed-loop activity, we applied a triangular pulse (200 ms) to the set point (Fig. 4B, top) and examined the time-dependent changes in the EMG signal as an outcome of the closed-loop activity (black line in Fig. 4B bottom). We then compared these with the EMGs reconstructed using each agIN (grey in Fig. 4B, bottom). In this simulation, we had three variables – the Ib-gain (blue in Fig. 4A), the displacement feedback gain (Ia-gain, orange in Fig. 4A), and the amplitude of set-point (red in Fig. 4A). We explored the optimal set of the three variables that provided the best match between the simulated and reconstructed EMG signals. In this procedure, the beta-fusimotor gain was fixed to illustrate the effects of changing a given variable.

The model consists of the muscle model using a modified Hill model (3), displacement feedback via muscle spindle Ia afferents, force feedback via tendon organ Ib afferents, positive feedback via the β-fusimotor pathway, and physical environments, as described previously (2). Except for the gain parameters, we used the same parameters as the original model (2) (Table S1). The physical environment (the hand of the monkey) was constructed as a one-link model with a damper whose rotational axis is the wrist (74).

$$J\ddot{\theta} = \tau - k_d\dot{\theta} + \tau^m$$

Where θ is the angle of the wrist, τ is the joint torque generated by the muscle, τ^m is the physical effect of the manipulandum. Moment of inertia J=6.38×10^{-3} kgm^2 (4), viscosity of wrist k_d=0.06 Nm s/rad were respectively set as fixed values based on the previous literatures. The physical effect of the manipulandum, τ^m, was modelled as a spring,

$$\tau^m = -k_s^m \theta,$$

and the spring constant k_s^m=1.0 Nm/rad was determined from its mechanical property.

Exploration of the optimal network parameter for simulating ag-IN generated EMGs

To find the Ib-gain, Ia-gain, and set-point amplitude (SP) that correspond to the reconstructed (Fig. 3) or actual EMG data (Fig. S5), we computed the RMSE (Root Mean Squared Error) between the simulated EMG signal (α-motoneuron output) and the reconstructed or actual EMGs, and searched for the Ib-gain and SP that minimize the RMSE. Here, the Ia-gain for optimization was set to 0.5×Ib-gain (Fig. 4G, J and Fig. S5G, J support that the optimal result is not sensitive to the Ia-gain value and that this assumption is reasonable). To determine the optimal parameters, we used the interior-point method with non-negative constraints. We used MATLAB 'fmincon' function for the implementation.

Examining the effect of Ib-gain, Ia-gain, and SP

To reveal the influence of the Ib-gain, Ia-gain, and SP on the simulated EMGs, we gradually changed each from their optimal values and performed a simulation for each parameter (Fig. 3C-E and Fig. S5C-E).

Characterization of the simulated EMG signals

To facilitate a comparison of simulated EMG across different Ib-gain, Ia-gain, and SP, which were systematically varied around their optimal values for the 22 agIN-muscle pairs, we characterized the simulated EMGs using two key metrics: normalized amplitude and sustaining duration of simulated EMGs (Fig. 4F–M and Fig. S5F–M). The normalized sustained duration was calculated as follows:

$$\frac{Ft\text{-}Mt_{opt}}{Ft_{opt}\text{-}Mt_{opt}} \quad (2)$$

where *Ft* is the time at which the simulated signal falls below half of the maximal amplitude, *Mt* is the time of the peak amplitude, and *opt* is the optimal value. The normalized amplitude was calculated as follows:

$$\frac{Max}{Max_{opt}} \qquad (3)$$

where *Max* is the peak amplitude, and *Maxopt* is the peak amplitude in the simulated EMGs obtained using the optimal parameter.

Figures

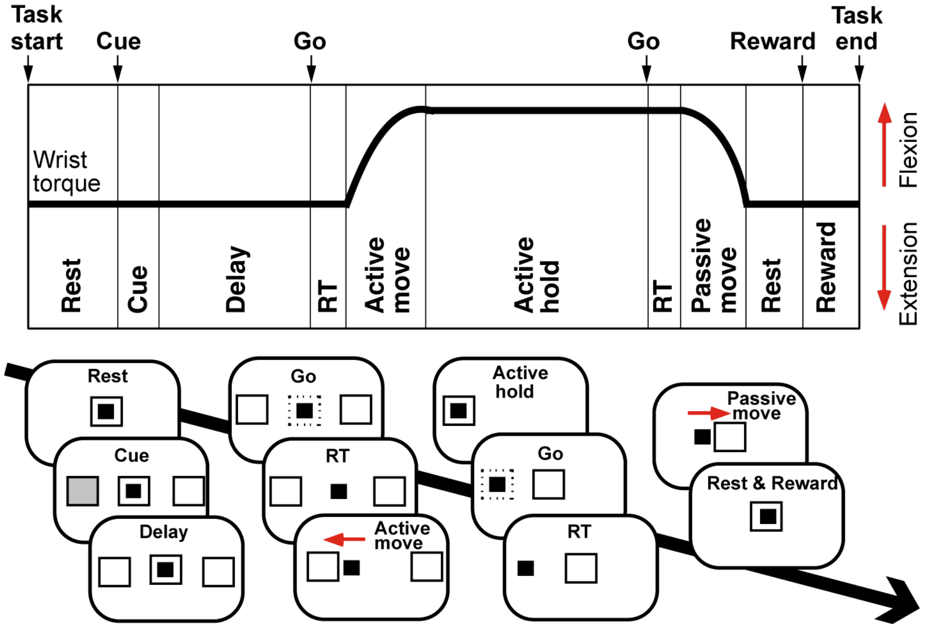

Fig. S1. Wrist flexion–extension task with an instructed delay period

Top: Schematic illustration of wrist torque measured during a single flexion trial. Bottom: Display instructions and presentation of monkey wrist movements. The black-filled square represents a moving cursor, the solid empty squares represent central and peripheral targets, and the grey-filled square represents the peripheral cue. The red arrows indicate the direction of movement. RT: reaction time. During the flexion trials, the active movement and hold epochs required a wrist

flexion movement, and the passive movement involved wrist extension (and vice versa for extension trials). See Methods for further details.

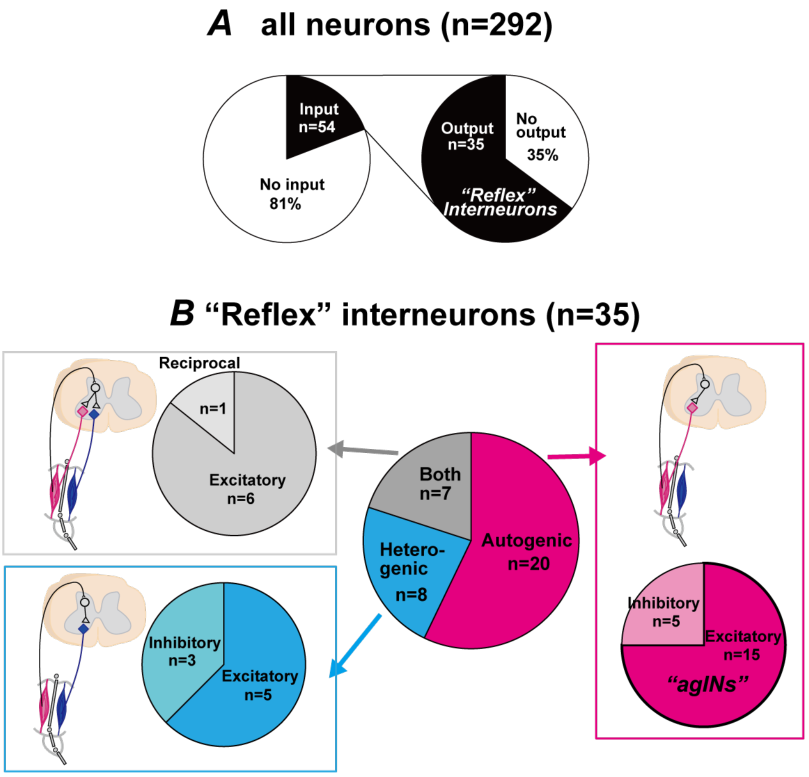

Fig. S2. Population of spinal interneurons recorded in this study.

A: A total 292 neurons were recorded from three monkeys, and 54 (13.8%) showed input from the DR nerve within a central latency of 1.5 ms. Among these, 35 exhibited a post-spike effect as revealed by spike-triggered averaging. B: Classification of reflex-related INs by their input-output pattern. Autogenic: The output is directed to the extensor muscles. Heterogenic: Output of the flexor muscles. Both: Output to flexor and extensor muscles. Excitatory: INs with post-spike facilitation (e.g., excitatory INs). Inhibitory: INs with post-spike suppression (e.g., inhibitory INs). The 15 excitatory INs exhibiting autogenic input-output patterns (agINs) are the main focus of this study.

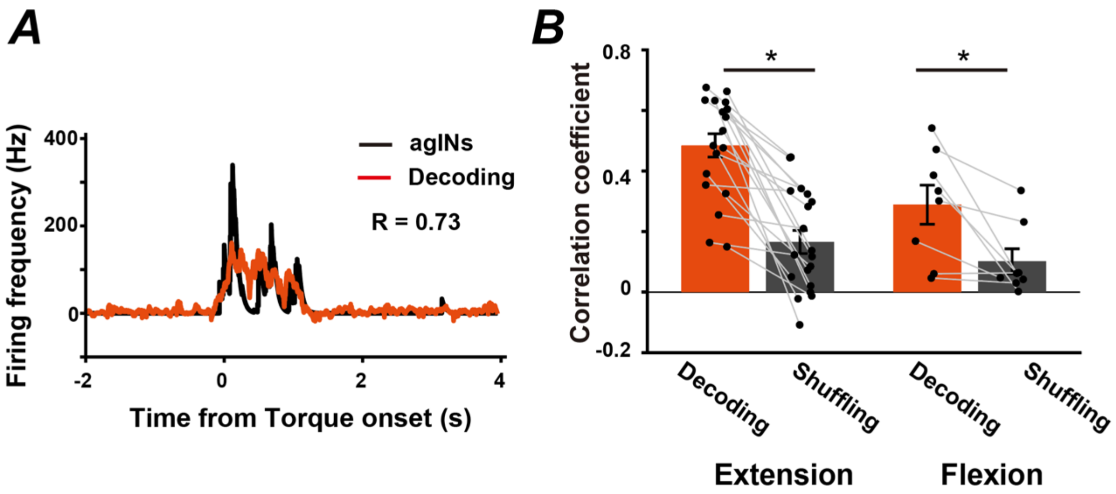

Fig. S3. Decoding the agIN's firing profiles by the EMG.

A: An example of the decoding of an agIN using the EMGs of the forelimb muscles of Monkey O. Black, actual activity; red, reconstructed activity. R: Correlation coefficient between observed and reconstructed activities. R = 0.73. B: Mean (±SEM) decoding accuracy pooled across INs. Correlation coefficients between the actual and reconstructed traces are presented (*p < 0.05, n = 19 for extension, n = 8 for flexion).

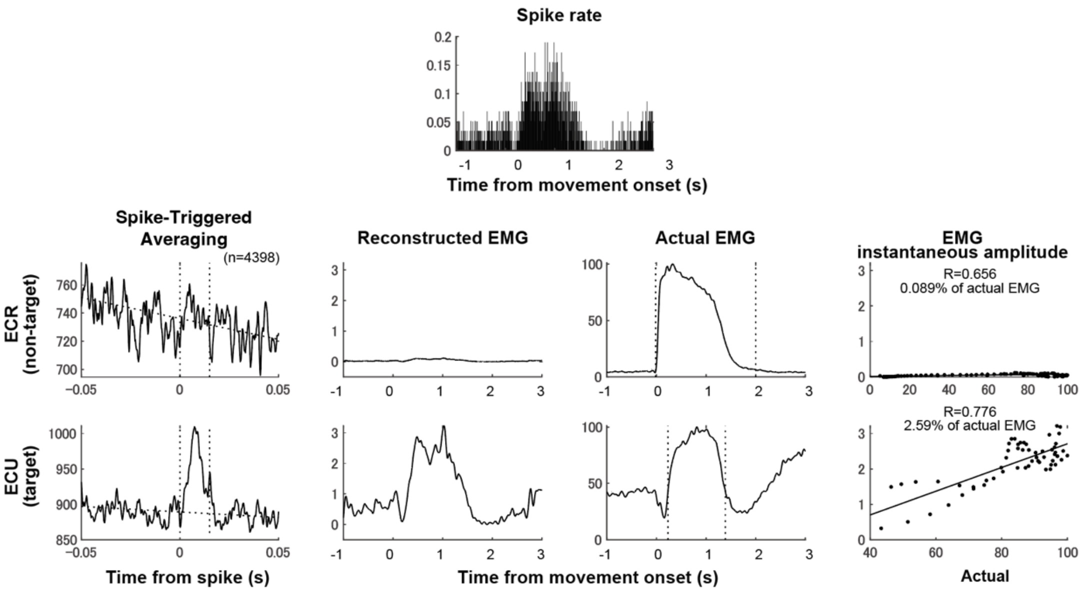

Fig. S4. Comparison of original and reconstructed EMGs of target and non-target muscles of a single agIN.

The second example of the actual- and the reconstructed EMG by convolution of the snippet with the post-spike effects on the target- and non-target muscles to the spike timing of single agINs. (A) Histogram of a single agIN aligned to the movement onset of extension trials (n=58). MO, movement onset. (B-E) Post-spike effect of the agIN shown in A (B), reconstructed EMG (C), actual EMG (D) and EMG instantaneous amplitude and linear regression line (E) for the non-target muscle. R, correlation coefficient. (F-I) Same format but showing results for the target muscle of agIN.

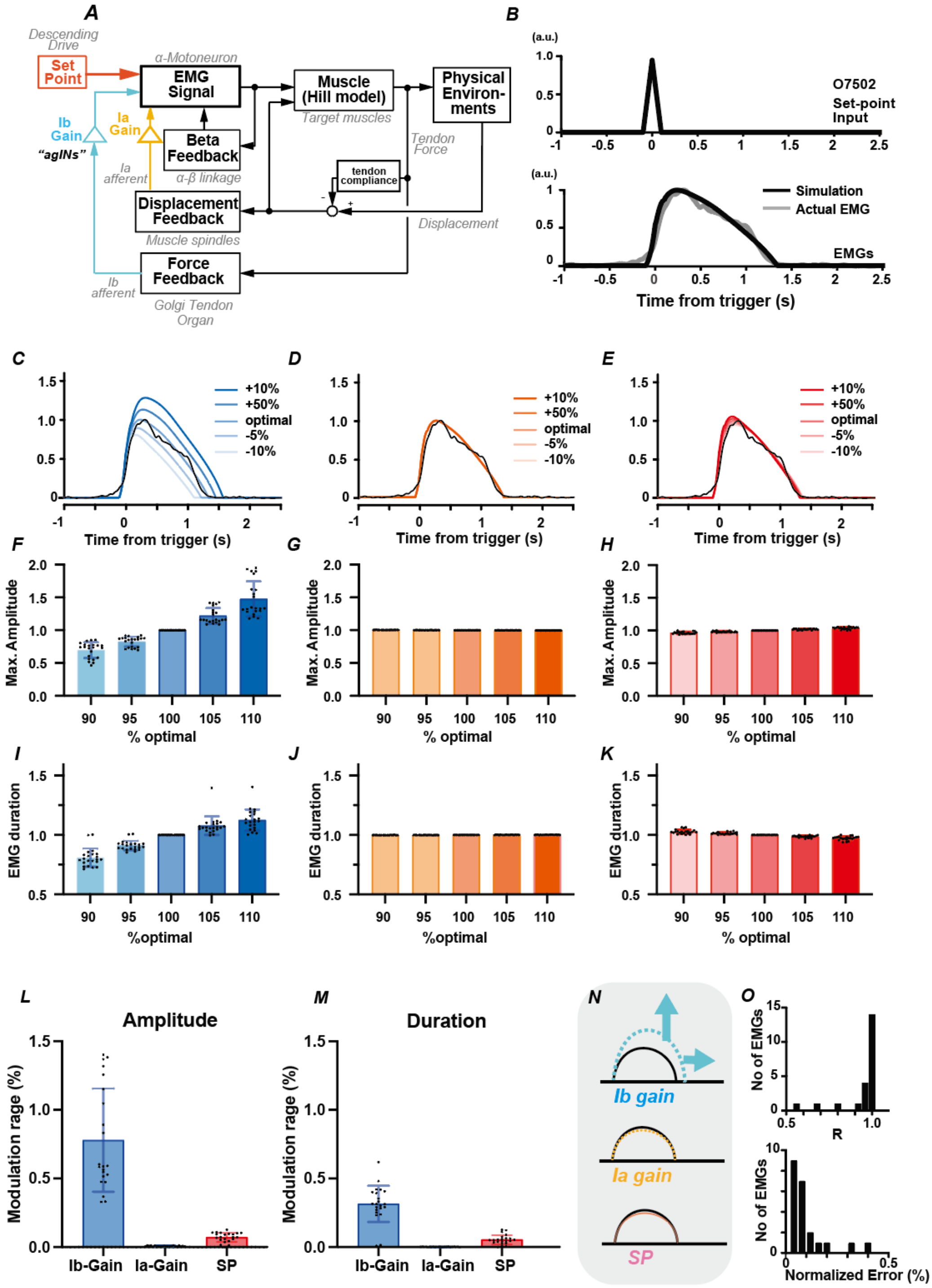

Fig. S5. Simulation for actual EMGs.

Result of simulation for the actual EMG. See the main text and legends for Fig. 4.

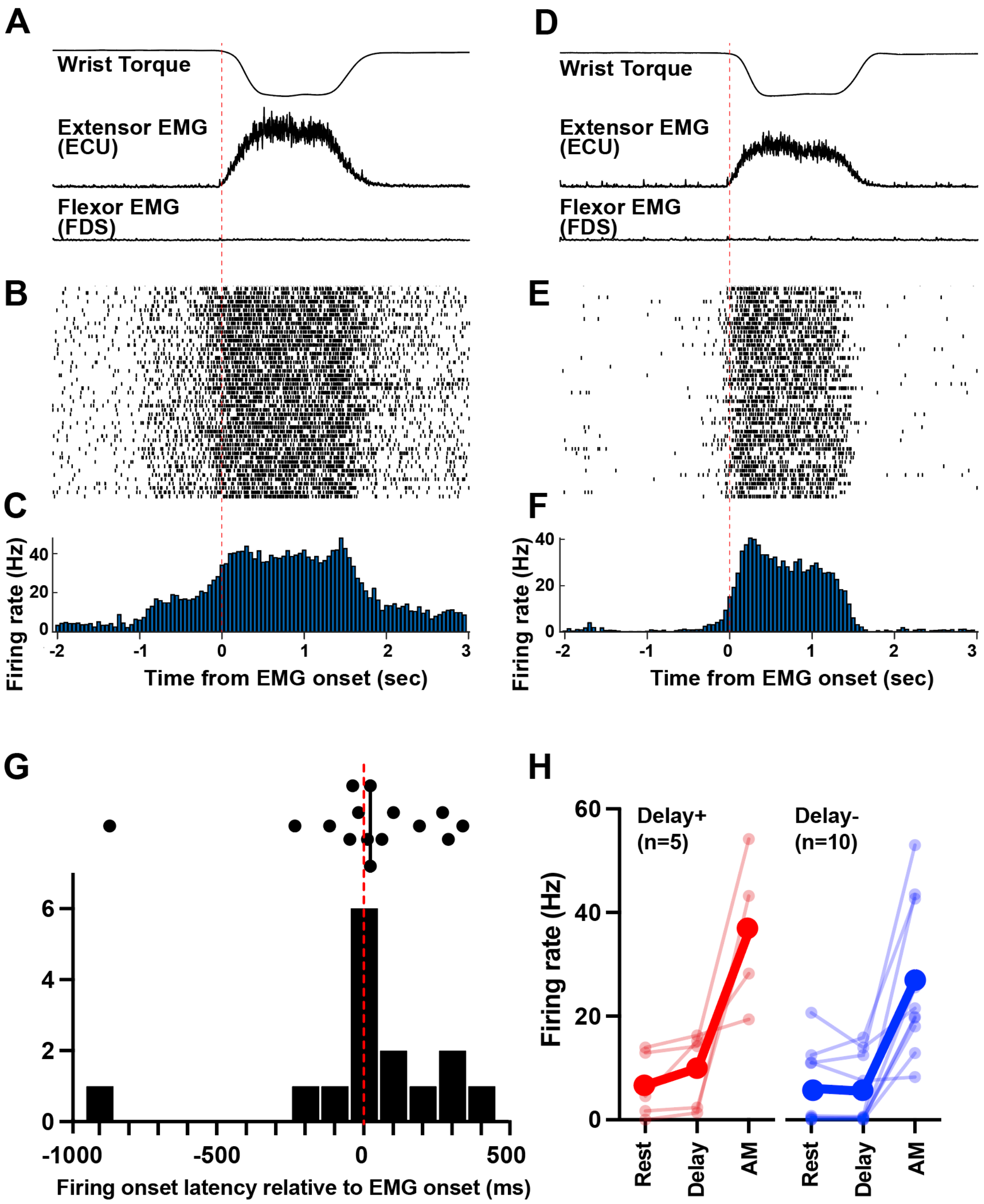

Fig. S6. Pre-EMG activity in agIN.

(A-F) Examples of agIN spiking activity are shown with simultaneously recorded EMG signals in wrist extension trials. (A,D) Average wrist torque and EMG of the extensor (ECU) and flexor (FDS) muscles. (B,E) Raster plots showing the activities of single agINs. (C,F) Peristimulus time histograms quantifying the modulation of agINs starting before the onset of EMG. Two examples

of agINs with dominant (latency = -870ms, A-C, n=50 trials) or subtle (latency = -10ms, E-F, n=50 trials) pre-EMG activity are shown. (G) Population summary of firing onset latency of agIN activity relative to extensor EMG onset. The histogram shows the distribution of firing onset latencies, defined as the time at which the peri-stimulus time histogram significantly deviated from baseline, relative to EMG onset. Each dot represents an individual agIN. The vertical black line indicates the median firing onset latency. Negative values indicate pre-EMG activity. (H) Comparison of agIN firing rates among epochs around movement onset. Mean firing rates of individual agINs are shown during the rest, delay, and active movement (AM) epochs. Each thin line represents a single agIN, and thick lines indicate the population mean. Neurons were grouped based on whether they exhibited a significant increase in firing rate from the rest to the delay epoch (Delay+, n = 5; Delay−, n = 10). For all panels, agIN activity and EMG signals were aligned to the onset of the extensor EMG, which is indicated by the red dashed line.

Tables

Table S1. Parameters used in the positive feedback model

1a Gain	0.5 × 1b Gain
1b Gain (optimal)	ag-IN: 2.17 (±0.42), actual EMG: 2.11(±0.30)
Beta Gain	0.5
Force feedback delay	40 ms*
Displacement feedback delay	10 ms*
Beta delay	20 ms*
Tendon compliance	0.05 mm/N*

* Same values as the model by Prochazka et al. (2).

SI References

1. J. A. Pruszynski *et al.*, Primary motor cortex underlies multi-joint integration for fast feedback control. *Nature* **478**, 387-390 (2011).
2. A. Prochazka, D. Gillard, D. J. Bennett, Implications of positive feedback in the control of movement. *J Neurophysiol* **77**, 3237-3251 (1997).
3. A. V. Hill, The heat of shortening and the dynamic constants of muscle. *Proceedings of the Royal Society of London. Series B - Biological Sciences.* **126**, 136-195 (1938).
4. J. A. Vilensky, Masses, centers-of-gravity, and moments-of-inertia of the body segments of the rhesus monkey (Macaca mulatta). *Am J Phys Anthropol* **50**, 57-65 (1978).
